# Comparative characterization of all cellulosomal cellulases from *Clostridium thermocellum* reveals high diversity in endoglucanase product formation essential for complex activity

**DOI:** 10.1186/s13068-017-0928-4

**Published:** 2017-10-23

**Authors:** Benedikt Leis, Claudia Held, Fabian Bergkemper, Katharina Dennemarck, Robert Steinbauer, Alarich Reiter, Matthias Mechelke, Matthias Moerch, Sigrid Graubner, Wolfgang Liebl, Wolfgang H. Schwarz, Vladimir V. Zverlov

**Affiliations:** 1 Department of Microbiology, Technische Universität München, Emil-Ramann-Str. 4, 85354 Freising, Germany; 2 Institute of Molecular Genetics, Russian Academy of Science, Kurchatov Sq. 2, Moscow, 123182 Russia

**Keywords:** Cellulosome, Synthetic complex, Synergistic hydrolysis, Cellobiohydrolase, Endoglucanase, Cellulase, Biomass degradation, Molecular docking

## Abstract

**Background:**

*Clostridium thermocellum* is a paradigm for efficient cellulose degradation and a promising organism for the production of second generation biofuels. It owes its high degradation rate on cellulosic substrates to the presence of supra-molecular cellulase complexes, cellulosomes, which comprise over 70 different single enzymes assembled on protein-backbone molecules of the scaffold protein CipA.

**Results:**

Although all 24 single-cellulosomal cellulases were described previously, we present the first comparative catalogue of all these enzymes together with a comprehensive analysis under identical experimental conditions, including enzyme activity, binding characteristics, substrate specificity, and product analysis. In the course of our study, we encountered four types of distinct enzymatic hydrolysis modes denoted by substrate specificity and hydrolysis product formation: (i) exo-mode cellobiohydrolases (CBH), (ii) endo-mode cellulases with no specific hydrolysis pattern, endoglucanases (EG), (iii) processive endoglucanases with cellotetraose as intermediate product (pEG4), and (iv) processive endoglucanases with cellobiose as the main product (pEG2). These modes are shown on amorphous cellulose and on model cello-oligosaccharides (with degree of polymerization DP 3 to 6). Artificial mini-cellulosomes carrying combinations of cellulases showed their highest activity when all four endoglucanase-groups were incorporated into a single complex. Such a modeled nonavalent complex (*n* = 9 enzymes bound to the recombinant scaffolding protein CipA) reached half of the activity of the native cellulosome. Comparative analysis of the protein architecture and structure revealed characteristics that play a role in product formation and enzyme processivity.

**Conclusions:**

The identification of a new endoglucanase type expands the list of known cellulase functions present in the cellulosome. Our study shows that the variety of processivities in the enzyme complex is a key enabler of its high cellulolytic efficiency. The observed synergistic effect may pave the way for a better understanding of the enzymatic interactions and the design of more active lignocellulose-degrading cellulase cocktails in the future.

**Electronic supplementary material:**

The online version of this article (doi:10.1186/s13068-017-0928-4) contains supplementary material, which is available to authorized users.

## Background

Due to the complex structure of plant cell walls, biomass-derived polysaccharides embody a rich tapestry of sugars and sugar compositions which are degraded by cellulases and other glycoside-depolymerizing enzymes. These enzymes can be described by three-dimensional structural analysis, sequence-based classification, substrate specificity, hydrolytic reaction mode, kinetic parameters, and product formation. Among carbohydrate-active enzymes, the CAZy database [1] classified 145 different glycoside hydrolase (GH) families as of August 2017, whereas cellulases are represented by 14 different GH families. The ill-defined term “cellulase” is generally taken to describe enzymes that depolymerize β-1,4-glycosidic bonds in β-glucans from cellulosic biomass. However, various cellulase types can be distinguished by their different modes of catalytic action. Exo-acting cellobiohydrolases hydrolyze the polysaccharide chain either from the reducing or non-reducing end, while endoglucanases cleave within the cellulose chain to generate new ends that are susceptible to subsequent hydrolysis by exoglucanase enzymes [2]. Binding of the enzyme to the substrate requires the presence of specific carbohydrate-binding modules (CBM) and sugar-binding residues on the enzyme surface and catalytic cleft.

According to the first description by Koshland, the catalytic reaction is retaining or inverting, depending on the nucleophilic attack at the glycosidic bond of the polysaccharide and the resulting stereochemistry of the anomeric carbon [3]. The measurement and classification of cellulase processivity is a daunting task, due to a variety of available assay techniques and a lack of established standards [2, 4]. Processivity can generally be defined as the average number of cleavages on the cellulose chain, before the enzyme dissociates from the substrate (catalytic rate coefficient *k*_cat_ divided by dissociation rate coefficient *k*_off_) [5]. The key differentiating factors among processivity of cellulases have been studied mainly in fungal cellulases and comprise the following: (i) The presence of loop structures to form a tunnel which covers the active site during the processive movement on the cellulose chain [6], (ii) the presence of certain CBMs linked to the catalytic core of an endoglucanase [7], and (iii) the presence of subsites for sugar binding and affinity [8]. Exo-acting cellulases known to hydrolyze the cellulose chains from the reducing ends are GH7 and GH48 enzymes, while enzymes processively acting from the non-reducing ends are GH9 and GH6 [5]. Other processive endoglucanases have also been reported for certain enzymes from the GH5 and GH9 families, such as Cel5H from *Saccharophagus degradans* [9] and Cel9I from *C. thermocellum* [10], respectively. In addition, cellulase actions are dictated by further structure–function–stability relationships, e.g., (N-terminal) extensions for stabilization of the catalytic core [11], the presence of specific ion binding sites for selective thermostabilization [12], or the influence of the quaternary structure on substrate specificity [13]. Instead of measuring the “apparent” processivity of cellulases, computational and structural modeling has been used to explain the “intrinsic” processivity of cellulases on a molecular level, as reviewed by [4, 14].

The cellulosomal complex of *Clostridium thermocellum* is one of the most efficient cellulase systems discovered to date [15]. This multi-modular enzyme system is based on the immobilization and co-localization of over 70 different proteins on a scaffolding structural protein, whereby different enzyme types act synergistically to efficiently degrade the polysaccharide into soluble sugars [16]. Interestingly, transcriptomic and proteomic analysis revealed that the cellulosome contains redundant sets of different cellulases and that regulation of their expression is a function of the substrate [17–19]. Nevertheless, the debate over why *C. thermocellum* (and other cellulolytic bacteria) express such vast and varied numbers of cellulases remains active.

To our knowledge, a comparative characterization of all β-1,4-glucanases present in the cellulosome has not been reported. In this study we characterize the product formation of 24 cellulases on different soluble and insoluble cellulosic substrates and β-1,4-glucans. Furthermore, a comprehensive comparison of activity profiles and product formation kinetics on model oligosaccharides and PASC (phosphoric acid swollen cellulose) is presented. We were able to differentiate between the apparent product spectra formed by GH5 and GH9 endoglucanases. To this end, a hydrolysis product pattern for Cel9D and four GH5 endoglucanases from sub-family 1 (Cel5O, Cel5B, Cel5G and Cel5L) was identified which distinguishes it from all other endoglucanase or cellobiohydrolase (CBH) hydrolysis patterns. Furthermore, we show that this new type of endoglucanolytic cleavage may have implications on the overall hydrolytic efficiency of synthetic (mini-)cellulosomes towards microcrystalline cellulose. The disparity in apparent processivity and substrate preference between glycoside hydrolases of family 9 (GH9) was supported by molecular docking experiments as well as sequence analysis revealing the presence of carbohydrate-binding modules (CBM) and sugar-binding moieties. Our data contribute to a deeper understanding of the cellulosomal cellulase system and may be of relevance for the design and engineering of more efficient enzyme mixtures for biomass degradation in the future.

## Methods

### Strains, media, and chemicals

*Clostridium thermocellum* (in the literature also referred to as “*Ruminiclostridium thermocellum”* [20]) DSM1237 was grown at 60 °C in prereduced GS-2 medium for liquid cultures containing 0.5% (w/v) cellobiose [21]. Recombinant *Escherichia coli* strains DH10B and BL21(DE) Star (Invitrogen, Carlsbad, USA) were used for cloning and protein expression, respectively. The cells were grown in Lysogeny broth containing 100 µg/mL ampicillin for pET21a(+) plasmids and 50 µg/mL kanamycin for pET24(+) plasmids. If not stated otherwise, chemical reagents were purchased by Sigma-Aldrich (Taufkirchen, Germany).

### DNA manipulation and synthesis

Preparation of chromosomal and plasmid DNA, endonuclease digestion, and ligation was carried out by standard procedures [22]. QIAprep Spin Miniprep Kit and PCR purification kit (Qiagen, Hilden, Germany) were used for purification of plasmids and PCR products. Restriction digests of DNA were done as recommended by the manufacturer (NEB, Ipswich, USA). Chemically competent *E. coli* DH10B cells were used for transformation with plasmid DNA.

Signal peptides were predicted by SignalP 3.0 server [23]. Genes without the signal sequence were amplified with oligonucleotide primers as listed in Additional file 1 and Phusion DNA Polymerase (NEB, Ipswich, USA) with chromosomal DNA from *C. thermocellum* DSM1237 as template. The synthesized genes *cel*124 (cthe_0435), *cel*9-44J, *cel*9K, and *cel*48S were optimized for *E. coli* codon usage by Eurofins (Ebersberg, Germany). The cellulosomal scaffolding protein CipA was synthesized in optimized *E. coli* codon usage and optimized DNA sequence, including eight cohesins Coh1-2, the carbohydrate-binding module CBM3, Coh3-8, and the C-terminal X-module from *C. thermocellum* WP_020458017.1 lacking Coh6 and Dockerin type-II. The resulting construct is referred to as CipA8 (see Additional file 2). The amplicons were digested and ligated in frame into the multiple cloning site of the plasmid pET21a(+). The correct sequence of all constructs was verified by resequencing (MWG, Ebersberg, Germany).

### Protein purification

For protein expression, the plasmids were transformed into *E. coli* BL21(DE) Star. The cells were grown at 37 or 20 °C and protein expression from pET21(+) or pET24(+) plasmids was induced by addition of 1 mM isopropyl-β-d-thiogalactopyranoside (IPTG) to an exponentially growing culture. After further growth at 37 °C for 4 h, the cells were harvested by centrifugation at 3440×*g* (Sorvall RC 6 +, Thermo Fisher, Waltham, USA) for 10 min at 4 °C.

The cells were resuspended in 20 mL lysis buffer (50 mM MOPS pH 7.3, 100 mM NaCl, 10 mM CaCl_2_, 20 mM imidazole) with the addition of lysozyme (AppliChem, Darmstadt, Germany) to a final concentration of 10 mg/mL and incubated for 30 min on ice. The cells were sonified twice with Sonifier UP 200S (Hielscher, Teltow, Germany) set at amplitude 60%, interval 0.25 and for 4 min. The supernatant after centrifugation (18,000 rpm, 20 min, 4 °C) was loaded onto an immobilized metal HisTrap affinity column (IMAC) (GE Healthcare, Munich, Germany) and eluted with 0.5 M imidazole, 50 mM MOPS pH 7.3, 100 mM NaCl, 10 mM CaCl_2_. The proteins were examined by sodium dodecyl sulfate–polyacrylamide gel electrophoresis (SDS-PAGE) and stained with Coomassie brilliant blue R-250. The protein concentration was determined spectrophotometrically by measuring the absorbance at 280 nm in a 5 M urea solution (Additional file 3). All protein preparations contained 20% glycerol (v/v) or sucrose and 0.2% sodium azide (w/v) and were proven to be stable on storage at − 20 °C. Table 1 summarizes all proteins analyzed in this study.

**Table 1:** Summary of the cellulosomal cellulases from *C. thermocellum* analyzed in this study

Enzyme	Locus tag Cthe	Glycoside hydrolase family	Sub-family	Catalytic mechanism^#^	References
Cel48S	2089	GH48		Inverting	[24]
Cel9K	0412	GH9		Inverting	[25, 26]
Cbh9A	0413	GH9		Inverting	[27]
Cel8A	0269	GH8		Inverting	[28]
Cel5E*	0797	GH5	4	Retaining	[29]
Cel5-26H	1472	GH5 GH26	25	Retaining/retaining	[30]
Cel9-44J	0624	GH9 GH44		Inverting/retaining	[31]
Cel9N	0043	GH9		Inverting	[10]
Cel9P	0274	GH9		Inverting	[17]
Cel9T	2812	GH9		Inverting	[32]
Lec9A	2761	GH9		Inverting	[17]
Cel124A	0435	GH124		Inverting	[33]
Cel5B	0536	GH5	1	Retaining	[34]
Cel5G	2872	GH5	1	Retaining	[35]
Cel5L	0405	GH5	1	Retaining	[17]
Cel5O	2147	GH5	1	Retaining	[36]
Cel9D	0543	GH9		Inverting	[37]
Cel9F	0543	GH9		Inverting	[38]
Cel9Q	0625	GH9		Inverting	[39]
Cel9R	0578	GH9		Inverting	[40]
Cel9U	2360	GH9		Inverting	[17]
Cel9V	2760	GH9		Inverting	[17]
Cel9W	0745	GH9		Inverting	[17]
Lec9B	0433	GH9		Inverting	[17]

* Protein was characterized without its carbohydrate esterase module

^#^Catalytic mechanism according to the CAZy database (http://www.cazy.org/)

### Native cellulosome and SM901 extract preparation

Well-grown cultures of *C. thermocellum* mutant SM901, also referred to as SM1 [41] were centrifuged twice (13,000 rpm, 20 min). Extracellular proteins were precipitated from the cell-free supernatant using saturated (NH_4_)_2_SO_4_ solution added to a final concentration of 60% (v/v). After overnight incubation at 4 °C the proteins were collected by centrifugation (15,000 rpm, 20 min, 4 °C). Supernatant preparations from mutant SM901 were resuspended in 50 mM MES, 0.1 M NaCl, 5 mM CaCl_2_, pH 6.0. Cellulosomal preparations from *C. thermocellum* DSM1237 were obtained by affinity digestion and purification method with modifications [42, 43]. Culture supernatant of 1 L well-grown *C. thermocellum* culture was spun down and incubated with 100 mg/L phosphoric acid swollen cellulose (PASC) overnight at 4 °C. Cellulosomes bound to amorphous cellulose were collected by centrifugation (13,000 rpm, 15 min, 4 °C) and resuspended in 20 mL dialysis buffer (50 mM Tris, 5 mM CaCl_2_, 5 mM DTT, pH 7.0). The suspension was incubated at 60 °C and dialyzed in a Slide-A-Lyzer cassette (MW cutoff 10,000 Da) against 2 L of dialysis buffer until the suspension was clear. A pure cellulosome preparation was obtained after spinning down hydrolysis debris. Purified enzymes were concentrated with Vivaspin 500 columns (Sartorius-Stedim, Göttingen, Germany) with a cutoff of 30 to 300 kDa. Sodium azide was added to the protein preparations in a final concentration of 0.02% (w/v).

### Substrates

Barley β-glucan was purchased from Megazyme (Wicklow, Ireland), Avicel, and carboxymethylcellulose (CMC) from Sigma-Aldrich (Taufkirchen, Germany). PASC was prepared from Avicel as described by Wood [44]. Substrates were used in enzymatic reactions at final concentrations of 0.5% (Barley β-glucan, CMC, PASC) or 1% (Avicel).

### Enzymatic assays

All enzymatic reactions were performed under standard reaction conditions at 60 °C in a total volume of 0.5 mL. The standard reaction buffer contained final concentrations of 0.1 M MOPS, pH 6.5, 50 mM NaCl, 10 mM CaCl_2_, and 2 mM of Tris(2-carboxyethyl)phosphine (TCEP) as reducing agent. The activity of single cellulases was determined with barley β-glucan, CMC, PASC, or Avicel under standard reaction conditions. The activity of complexed cellulases was determined with Avicel (0.25% final concentration) with a standard enzyme load of 2 µg/mL. The enzyme kinetics were performed with 2.5% Avicel and 2 µg/mL of the enzymes. To avoid inhibition of the complexed cellulases by cellobiose, β-glucosidase (TTP0042) from *Thermus thermophilus* [45] was added to a final concentration of 6 µg/mL. Reducing sugar ends released from the substrates were quantified in triplicates using 3,5-dinitrosalicylic acid method [46]. One enzymatic unit liberates 1 µmol of glucose equivalent per minute.

### Binding affinity studies on CipA8 and gel mobility shift assay (EMSA)

Single cellulases were bound to recombinant CipA8 by titrating different stoichiometric ratios of 1:2, 1:4, 1:6, 1:8, and 1:10 (CipA8:enzyme). The assays were performed in 30 µL reaction volume with 10 mM CaCl_2_ and 0.05 nmol of scaffolding protein CipA8. After 1 h of incubation at room temperature, the dockerin–cohesin interaction resulted in molecular shifts of the unbound cellulases, as visualized by gel mobility shift assay (EMSA) on 6% native gel. Non-complexed CipA8, single enzymes, and native cellulosome were used as standards.

### Complex assembly

Cellulase complexes were assembled in gel filtration buffer (50 mM MOPS pH 7.3, 0.5 M NaCl, 20 mM CaCl_2_) for 1 h at room temperature. The complexes were assembled with a fixed concentration of the structure protein with 8 cohesins type-I and an equimolar amount of cellulases to the number of cohesins. These complexes were purified from non-complexed proteins by size-exclusion chromatography on a Superdex 200 10/300 GL column (GE Healthcare, Little Chalfont, UK) and equilibrated with gel filtration buffer. Size-exclusion chromatography was carried out on an ÄKTA Purifier (GE Healthcare, Munich, Germany). The column was developed with the same buffer at a flow rate of 0.5 mL/min. Fractions of 1 mL were collected and concentrated with Vivaspin 500 columns with a cutoff of 50 kDa. Protein concentration was determined by the BCA method [47] using bovine serum albumin as a standard.

### Product analysis

The kinetics of product formation were studied on PASC and β-1,4-gluco-oligosaccharides (cello-oligosaccharides) from DP2 (cellobiose) to DP6 (cellohexaose) by thin-layer chromatography. Aliquots were taken at different time points during an enzymatic reaction, and the enzyme was inactivated by incubation at 95 °C for 15 min and subsequently stored at − 20 °C for further analysis. One to 5 µL of the aliquots was spotted on TLC silica gel 60 aluminum plates (Merck, Darmstadt, Germany) using acetonitrile/water (80:20, v/v) as the mobile phase. A mixture of DP1—DP6 cello-oligosaccharides was used as standard. Detection was performed according to De Stefanis and Ponte [48], documentation and density plot calculation was performed with ImageJ (http://imagej.net/). Glucose tetramer type B (G4G3G4G) and type C (G4G4G3G) were analyzed using a high-performance anion-exchange chromatography with pulsed amperometric detection (HPAEC-PAD) on an ICS 3000 Dionex chromatography system with a CarboPac PA1 column (4 × 250 mm) and a PA1-precolumn (4 × 50 mm). The column temperature was set to 30 °C and the injection volume was 25 µL at a flow rate of 1 mL/min. The eluent gradient for analyte separation was 7.5 mM sodium acetate with 100 mM NaOH at 0 min and increased linearly up to 100 mM sodium acetate with 100 mM NaOH at 67.5 min. After each run, the washing step consisted of 650 mM sodium acetate during 4 min and equilibration with 100 mM NaOH for 16.3 min. Carbohydrate detection based on the waveform “standard carbohydrate quad” was set to 1 Hz. Samples were diluted by factor 10 with Milli-Q water before analyzing the polysaccharide hydrolysates by HPAEC-PAD. All oligosaccharides were purchased at Megazyme, Bray, Ireland.

### Structural sequence alignments and molecular docking

Multiple sequence alignments were performed with T-Coffee (http://tcoffee.crg.cat/) [49] and ESPript 3 (http://espript.ibcp.fr/ESPript/ESPript/) [50]. The sequence similarity tree was visualized with Mega 5.2 [51]. Structure prediction was performed using RaptorX (http://raptorx.uchicago.edu/), and models obtained were visualized as surface plots and amino acid overlay with the Visual Molecular Dynamics program. In silico docking experiments with cellohexaose and selected cellulases were performed with AutoDock Vina (version 1.1.2) [52] using the following procedure: Water molecules and ligands were deleted manually and structural alignments were performed using MultiSeq in the Visual Molecular Dynamics program, resulting in aligned pdb files. Aligned molecules were rotated with PyMOL (x55, y20, z-24) and saved separately. Polar hydrogens were added using AutoDockTools (version 1.5.6) [53], macromolecule was chosen under flexible, residues were selected, and rotational bonds were defined. AutoDock was performed with flexible residues (exhaustiveness 24), and the results were loaded in chimera and saved from ViewDock and converted with OpenBabel (automated bonding disabled). All input molecules were joined into a singular output molecule. Proteins and the cellohexaose sugar substrate were visualized as surface model representation.

## Results

### Characterizing the cellulosomal cellulases

According to genome sequence analysis and to proteomics data of the extracellular cellulosomal complex of *C. thermocellum* [17, 18, 33, 54], in total 24 cellulase-encoding genes were selected for subsequent enzyme characterization (Table 1). The ORFs encoding putative cellulolytic proteins were subjected to PCR-cloning or gene synthesis. Enzyme preparations were obtained by heterologous expression without the predicted N-terminal signal peptide sequence and subsequent His-tag purification (purified enzymes are summarized in Additional file 3). As the proteins were expressed with an intact type-I dockerin binding module, the binding capacity of each protein was tested on the recombinant scaffolding protein CipA8 with eight single cohesin modules. All tested proteins assembled with CipA8 via cohesin–dockerin interaction. However, the molar ratio of full stoichiometric binding varied for each enzyme (see Additional file 4).

**Fig. 1:**
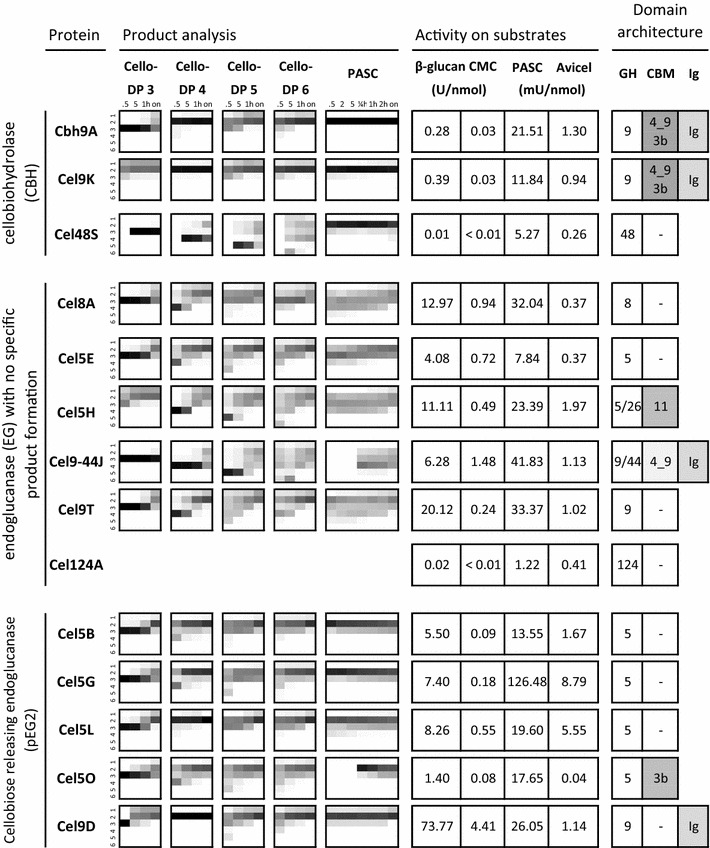
Comparison of all β-1,4-glucanases from the *C. thermocellum* cellulosome (listed in first column). Second column: Intermediate and final product analysis of different cellulases on various cello-oligosaccharides and PASC as substrate. The arrays show the oligosaccharide products (degree of polymerization ranging from glucose DP 1 to cellohexaose DP 6) on the *Y*-axis and the kinetic product shift over time (*X*-axis). The sugar amount detected by thin-layer chromatography is depicted as heat map representation, with relative intensities of the sugar products ranging from 1% (light gray) to 100% (black). Explanations of time points: 0.5 = 0.5 min; 2 = 2 min; 5 = 5 min; ¼ h = 15 min; 1 h = 60 min; 2 h = 120 min; on = overnight incubation. Empty fields (white) indicate that no products were formed, or products were below the detection limit of thin-layer chromatography. The pattern of protein CtCel124 is not shown due to its low activity. Third column: Activity of recombinant cellulases on various substrates (average values from triplicate measurements) at optimal cellulosome activity parameters (60 °C, pH 5.8; see Additional file 6). Fourth column: presence of glycoside hydrolase (GH) families, carbohydrate-binding modules (CBM), and Ig-like modules (Ig). For continuation of this figure, please see Fig. 2

**Fig. 2:**
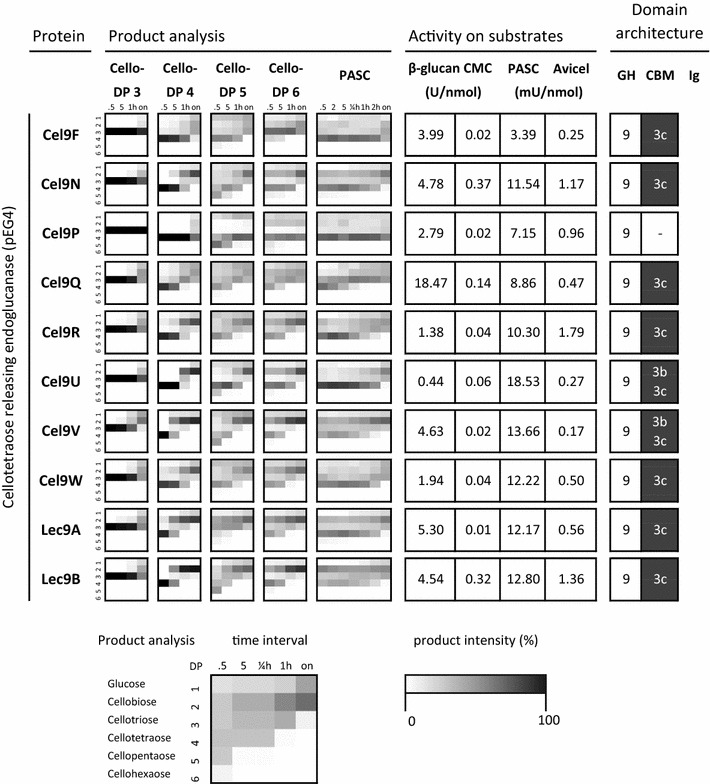
Comparison of all β-1,4-glucanases from the *C. thermocellum* cellulosome (continuation of Fig. 1)

In order to identify true β-1,4-glucanases, the degradation capability of glucose tetramer type B (G4G3G4G) and type C (G4G4G3G) was determined by HPAEC-PAD. Only Cel5-26H was specifically cleaving the β-1,3-glycosidic bond, whereas the other enzymes had no detectable activity on this type of glycosidic bond (see Additional file 5). Concomitantly, the products formed from model cello-oligosaccharides (cellotriose to cellohexaose), and activity on various cellulosic substrates were assessed (Figs. 1, 2). Unmodified substrate preparations were amorphous PASC and insoluble Avicel. In order to distinguish exo- from endo-acting cellulases, various β-glucan backbones were tested either with mixed-linkage β-1,3/1,4-glucan (barley) or side chain-modified CMC. Cleavage of these substrates is an indication for endo-acting cellulases which hydrolyze randomly at the β-1,4-linkages of the polysaccharide chain. In contrast, exo-acting cellobiohydrolases thread the cellulose molecule from its free cellulose chain end through a tunnel built by loop structures around the active site. Modified and mixed-linkage β-glucans block the enzymes’ processive activity by steric hindrance. Hence, significant activity is only observed on unmodified cellulose. The specific enzyme activities (µmol of reducing sugar ends per minute and per nmol of protein) were obtained under the optimal conditions for cellulosome activity (at 60 °C and pH 5.8; see “Methods” section and Additional file 6).

The substrate preference and sugar product spectrum of the cellulosomal cellulases vary substantially, regardless of enzyme family and module architecture. As expected, for the CBHs Cbh9A, Cel48S, and Cel9K no or very weak activities on modified substrates were observed, whereas on PASC substantial product formation was found. In contrast, Cel9D, Cel9-44J, Cel8A, and Cel5E were most active on CMC. Other proteins like Cel5L and Cel5G released the highest amount of reducing sugar ends on microcrystalline cellulose.

The apparent hydrolysis pattern of these enzymes was further studied on various cello-oligosaccharide standards and PASC with TLC over time (Figs. 1, 2). A suitable enzyme dilution was chosen to visualize the presence of all intermediate products formed during the hydrolysis reaction. To this end, we were able to identify four different product patterns. As expected, CBHs (exo-acting from the sugar ends) released specifically cellobiose as the only product over time (Cel48S, Cel9K, and Cbh9A). In contrast, endo-acting β-1,4-glucanases showed a more diverse product pattern. On PASC, the apparent random cleavage mode of non-processive endoglucanases (EG) is indicated by the formation of diverse cello-oligosaccharides and longer chain dextrins like cellopentaose (DP ≥ 5) at the beginning of the hydrolysis reaction with no preferred product at any time. This pattern is found with different GH family proteins such as Cel8A, Cel5E, Cel5-26H, Cel9-44J, and Cel9T. After prolonged incubation times (overnight), the final products are mainly cellobiose and cellotriose (DP 2 to DP 3). In contrast, processively acting endoglucanases are characterized by specifically cleaving off short-chain oligosaccharides of defined length (DP 2 or 4) at the beginning of the hydrolysis on PASC. This can be interpreted as an internal cut into the cellulose chain followed by a processive cleavage of even-numbered short cello-oligosaccharides before the enzyme falls off. Two different groups of processive endoglucanases can be distinguished, depending on the main product formed during hydrolysis: pEG4 and pEG2.

The cellotetraose-type processive endoglucanase (pEG4) group demonstrates cleavage and release of defined cello-oligosaccharides with DP 4 as intermediate product at the beginning of the hydrolysis reaction on the tested substrates. All the members of this group belong to glycoside hydrolase family 9. In cellobiose-type endoglucanases (pEG2), only cellobiose and small amounts of cellotriose as intermediate and final products were observed, e.g., all members of GH5 sub-family 1 (Cel5B, Cel5G, Cel5L, and Cel5O). Interestingly, the pEG2 hydrolysis pattern is also demonstrated by Cel9D, which resulted in cellobiose and a small amount of glucose as the only and final degradation products. This result was confirmed by the hydrolysis products from cello-oligosaccharides as substrate, which also produced cellobiose as major degradation product, whereas glucose was released to a lesser extent (Figs. 1, 2).

### Role of endoglucanase processivity in synthetic protein complexes

**Fig. 3:**
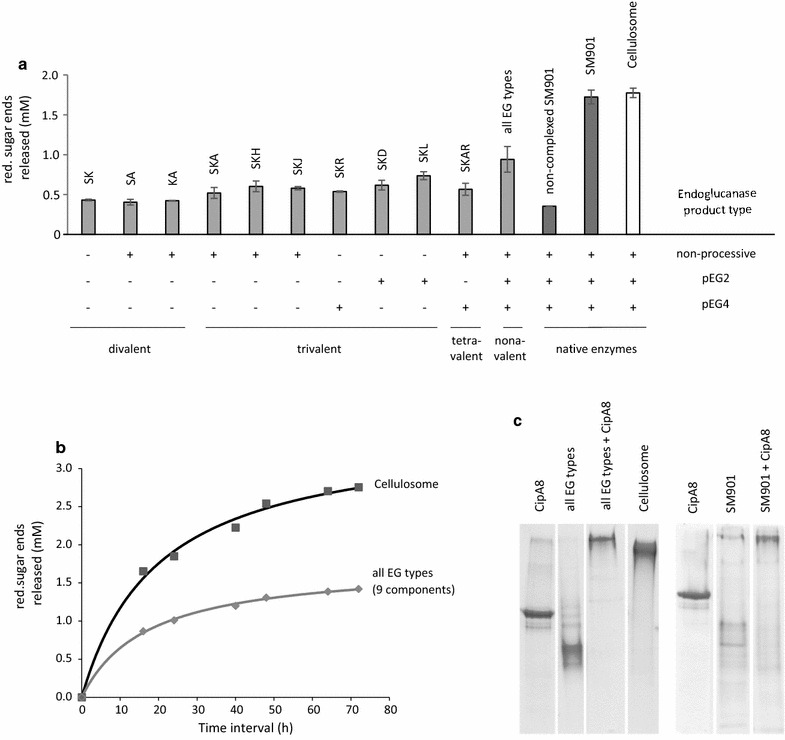
Hydrolytic efficiency of multi-enzyme complexes on Avicel as substrate. **a** End-point activity of 1 µg of the enzymes on 0.25% Avicel after 42 h in dependence of the endoglucanase functions present in the complex. The bars represent amount of reducing sugar ends (glucose equivalents) as average values from biological replicates (at least duplicate measurements with standard deviations represented as × 1 SD). The endoglucanase product pattern present (+) or absent (−) in the complex was non-processive (EG) or processive with cellobiose (pEG2) or cellotetraose (pEG4) as intermediate or main product. The complex “all EG types” consists of 9 different enzymes, whereas each cellulase function is present in this complex (cellobiohydrolases, non-processive endoglucanases, members of pEG2 and pEG4, respectively). Each of the complexed mixtures comprised equal stoichiometric loading and statistical distribution of eight single enzymes on CipA8 by cohesin–dockerin protein interaction. The enzyme complexes were purified by gel filtration to exclude the impact of unbound single cellulases. As controls, the activity of complexed and non-complexed enzyme extracts of *C. thermocellum* mutant SM901 [41] is shown together with the native cellulosome. Abbreviations: Cel8A (A), Cel9D (D), Cel5-26H (H), Cel9-44J (J), Cel9K (K), Cel5L (L), Cel9R (R), and Cel48S (S). **b** Enzyme kinetics of cellulosomal complexes on 2.5% Avicel. **c** Electrophoretic mobility shift showing the binding capacity of recombinant scaffolding protein CipA8 made possible by its eight cohesin binding modules. Complex formation by cohesin–dockerin interaction is visible by up-shifted protein bands in the native gel. 10 µM of CipA8 was titrated with 80 µM of a nonavalent cellulase mixture (all EG types + CipA8) for statistically binding all free cohesin modules. As another control, the SM901 enzyme extract was also completely bound (SM901 + CipA8). The 6% native PAGE gel was stained with Coomassie R-250

The presence and selective attachment of single enzymatic functions to the scaffolding protein has been discussed to be the key factor for effective cellulose degradation by the native cellulosome and synthetic multi-enzyme complexes [43, 55]. The discovery of different processivity groups of cellulases (Figs. 1, 2) prompted us to construct di-, tri-, and tetravalent mini-cellulosomal complexes to test their efficiency and synergism on microcrystalline cellulose: Different combinations of endo- and exo-active cellulases were bound to the scaffolding carrier protein CipA8 in equal stoichiometric loadings via the specific dockerin–cohesin protein–protein interaction. Upon loading of the scaffolding protein to saturation (all binding positions are bound by single cellulases), the high-molecular weight fractions were separated from unbound single cellulases by size-exclusion chromatography and pooled. The complex activity resulted in the release of soluble reducing sugar products from the insoluble substrate Avicel (Fig. 3a). As a result, after 2 days of incubation at 60 °C, divalent cellulase combinations of endo/exo as well as exo/exo components (basic complex with cellulases SK, KA, and SA, meaning Cel48S/Cel9K, Cel9K/Cbh9A, and Cel48S/Cbh9A complexes, respectively) showed the lowest activities with less than 500 µM reducing sugar end products per reaction, as compared with trivalent complexes comprising two CBH enzymes (one from the reducing sugar end and one from the non-reducing end-type, Cel48S and Cel9K, respectively) and one endoglucanase. To further analyze the impact of the type of endoglucanase incorporated in the complex, we further compared the presence of non-processive endoglucanases (complex SK with Cel5-26H) with processive ones (complex SK with Cel9R, Cel9L, and Cel9D, respectively). Interestingly the complexes containing the processive endoglucanases Cel5L (complex SKL) gave the best result (up to 736.6 µM) from all trivalent mini-cellulosomal complexes. Even a complex of four different enzymes (SKAR) including two different endoglucanase functions (non-processive and cellotetraose-releasing endoglucanase, whereas a pEG2-type was missing) did not result in higher productivity (566.2 µM).

In order to analyze the influence of more endoglucanase functions on a complex, we designed a fully synthetic cellulosomal nonavalent complex (“all EG types”) containing 25% of Cel48S and Cel9K, Cbh9A, Cel8A, Cel9Q, Cel9T (each 12.5%, corresponding a stoichiometric binding to one cohesin module) and a mixture of Cel5G, Cel9R, and Cel9-44J (each 4.2%) which most closely resembles the cellulase composition of the native cellulosome complex. The fully recombinant enzyme mixture (termed “all EG types”) contains all different classes of endoglucanase functions and showed on average 52.6 ± 1.4% of the activity of the native cellulosome enzyme preparation from *C. thermocellum* on 2.5% microcrystalline cellulose (Fig. 3b). The single enzyme components as well as the native enzyme mixture from *C. thermocellum* mutant SM901 assembled with recombinant scaffolding protein CipA8 to form enzyme complexes, whereas the stoichiometric binding capacity equals 1:8 (CipA8: single enzyme ratio) (Fig. 3c).

### Comparative sequence analysis and structural modeling

In order to predict certain sequence signatures that trigger the processive status of the endoglucanases, the module architecture, the presence of carbohydrate binding and other modules as well as tertiary/secondary structure prediction and sugar-binding moieties was compared. The multiple sequence alignment analysis of all 24 full-length protein sequences (including catalytic core and adjacent modules like CBMs, immunoglobulin-like modules, and others) could not differentiate between the apparent processivity status and the product specificity between the cellulosomal endoglucanases (data not shown). Noteworthy, this is also the case for the subset of cellulases belonging to GH9 which represent the majority of all cellulosomal cellulases (13 out of 24 cellulases in total).

**Fig. 4:**
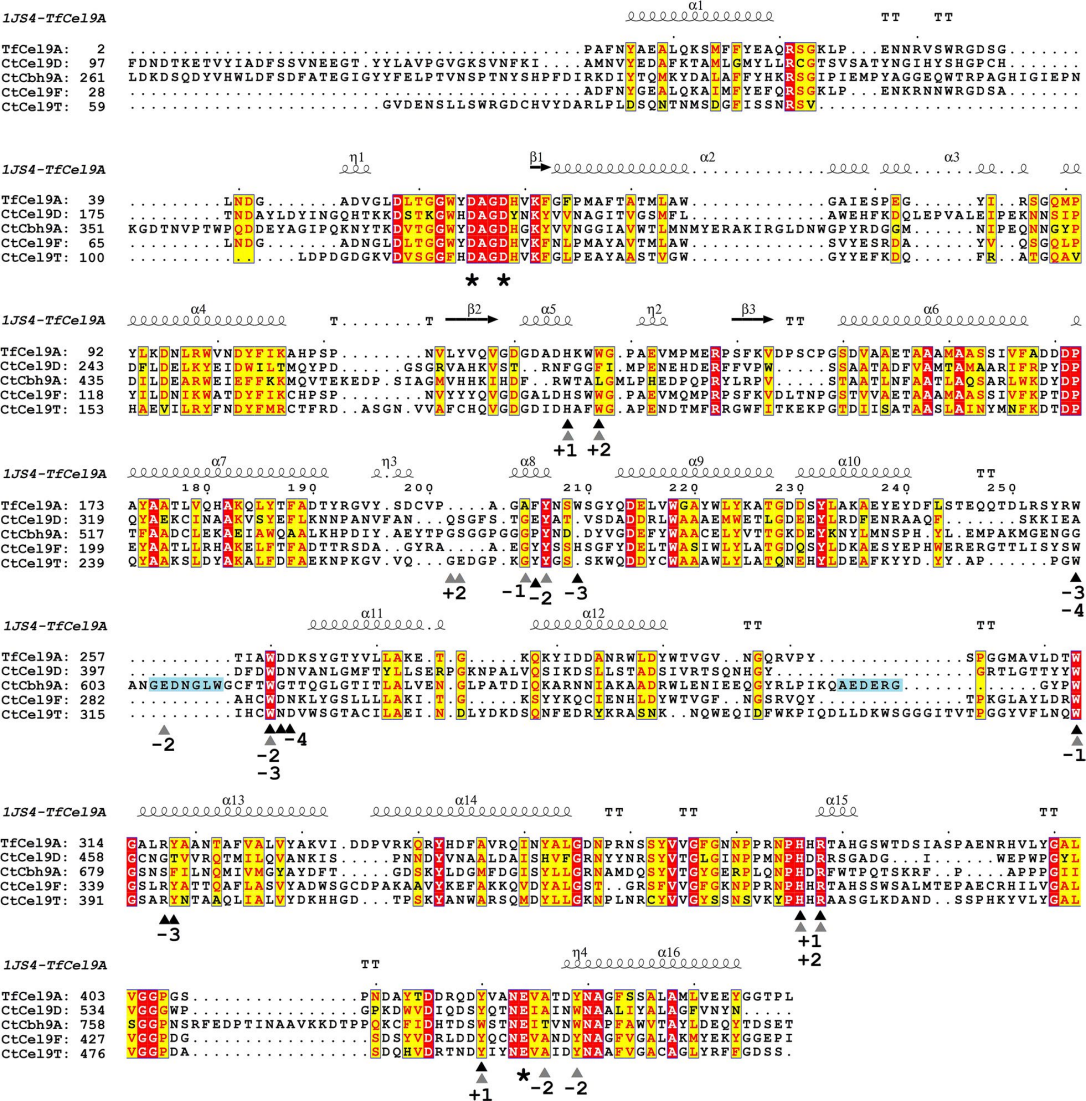
Structure-based multiple sequence alignment of GH9 family catalytic modules of four *C. thermocellum* cellulases: Cel9D, Cbh9A, Cel9T, and Cel9F. α-Helices (α- and *η*-helices), β-sheets, and loops in Cbh9A are indicated and numbered above the sequences as squiggles and arrows, respectively. Strict α-turns are indicated with TTT, strict β-turns with TT. The catalytic triad in the active sites is indicated with asterisks. Amino acids of the endoglucanase TfCel9A from *Thermobifida fusca* known to be involved in substrate-binding [59, 60] are shown as black triangles, those identified from cellobiohydrolase Cbh9A [56] are marked as gray triangles. The numbers below indicate the corresponding cello-oligosaccharide positions reported to interact/bind. Carbohydrate positions + 1 and + 2 are the expected product sites. Loop regions conferring exo-activity of Cbh9A are highlighted in light blue [56]

**Fig. 5:**
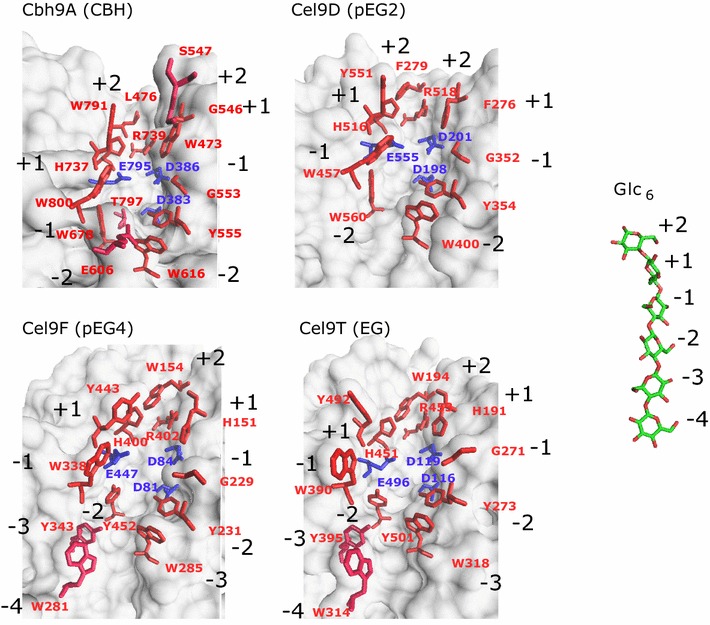
Structural comparison of four different catalytic clefts from glycoside hydrolase GH9 cellulases. The figure depicts cellobiohydrolase Cbh9A (PDB structure 1RQ5), processive endoglucanase pEG2 (Cel9D, PDB 1CLC), pEG4 (Cel9F, predicted structure), and non-processive endoglucanase Cel9T (PDB accession 2YIK) as gray surface plots and their corresponding sugar-binding moieties (red sticks) and catalytic triad (in blue). Cellohexaose (Glc_6_) was taken from PDB 7CEL. Numbers in black depict cello-oligosaccharide positions (+ 2 to − 2) according to the nomenclature for sugar-binding subsites [61] in the catalytic cleft from protein–ligand interaction data for Cbh9A according to [56], Cel9T [57], Cel9A from *T. fusca* [59] and the structural sequence alignment (Fig. 4)

Structure-based multiple sequence alignments and molecular modeling analysis of representative GH9 catalytic modules with different product spectra were performed: cellobiohydrolase Cbh9A [56], non-processive endoglucanase Cel9T [57], and the processive endoglucanase Cel9D [58]. The catalytic module of Cel9A (formerly called E4) from *Thermobifida fusca* (formerly known as *Thermomonospora fusca*) was chosen as it has been intensively characterized and as it shares relatively high sequence identity to Cel9F (57.2%) and Cel9T (35.9%), respectively [7, 59] (Fig. 4). The comparative analysis revealed 12 α-helices forming the (α/α)_6_-barrel fold typical for GH9 catalytic modules and amino acid residues that may be involved in substrate-binding, according to available structural data [56–59] and molecular docking simulations (Fig. 5). The active site comprises the conserved catalytic triad of the nucleophile/base (two aspartic acid residues in the DAGD-motif) and glutamic acid as catalytic proton donor. Sugar-binding moieties that are conserved in the sequence alignment share aromatic properties (tyrosine Y, tryptophan W) or are amino acids with electrically charged side chains (arginine R, histidine H, aspartic acid D, and glutamic acid E). The number of predicted substrate-binding residues varies between the Cbh9A with 14 residues, followed by Cel9F and Cel9T (12 residues each) and Cel9D comprising 10 interaction partners. Subsites G553, Y555, W616, W678, H737, and R739 of cellobiohydrolase Cbh9A are conserved among the compared structures covering the interactions of carbohydrate-binding positions + 2 to − 3 relative to the glycosidic linkage cleaved, while W473, L476, G546, S547, and T797 are unique sites with binding to position + 2 to − 2 cello-oligosaccharides. One of two loop regions that confer exo-activity in Cbh9A comprises E606 as another binding residue. In contrast, aromatic residues needed for interaction with larger sugars at position − 3 and − 4 were found to be present in endo-mode acting enzymes only, but are absent in Cbh9A and Cel9D. As putative binding residues we identified the residues W281, Y343 for Cel9F and W314, Y395 for Cel9T, respectively. Both aromatic amino acids are strictly conserved in this particular position among all other cellulosomal endoglucanases of family 9 (data not shown). In similarity to Cbh9A, the cellobiose-releasing processive endo-acting cellulase Cel9D lacks these aromatic residues binding to cello-oligosaccharides at positions − 3 and − 4, whereas unique aromatic sugar-binding residues are predicted, e.g., F276 instead of histidine at subsite + 2 and W560 instead of tyrosine at subsite − 2. Again, all other endoglucanases including Cel9T and Cel9F share conserved histidines or tyrosines at these particular positions as a common feature.

## Discussion

The recalcitrant nature and heterogeneous physical structure of cellulose selects for a varied arsenal of enzymatic machinery to efficiently degrade this kind of biomass. The native cellulosome of *C. thermocellum* is a model for co-localization of single enzymes on carrier proteins for synergistic activity on crystalline cellulose. The steric proximity of different enzyme classes seems to be the key feature of the cellulosomal system, inspiring researchers to systematically study and develop modified in vitro cellulase complexes [16, 43, 55, 62, 63]. From all of the more than 70 identified enzyme components identified via genome, transcriptome, and proteome analysis, 24 different enzymes are associated with the scission of cellulosic β-1,4-glycosidic bonds by exhibiting β-1,4-glucanohydrolase activity [17–19, 54]. Despite hydrolyzing an identical chemical bond, these cellulases are generally distinguished by their protein fold, mode of hydrolysis, and substrate specificity, as documented in the CAZy online database of glycoside hydrolase family proteins (http://www.cazy.org/). The cellulases present in the cellulosome of *C. thermocellum* are found in five different GH families (Table 1), namely families 5, 8, 9, 48, and the recently identified family 124 [1, 33]. Although all single enzymes have been reported before (Table 1), the lack of standardized experimental conditions (enzyme and substrate loading) has hindered any meaningful inter-laboratory comparisons of the available biochemical data. In this study activity, parameters like temperature, buffer, and pH were chosen in accordance with the optimum reaction conditions of the native cellulosome (Additional file 6).

Analysis of intermediate product kinetics and product ratios was employed to distinguish different processivity groups with the aid of thin-layer chromatograms of all 24 cellulosomal cellulases. This approach allowed for qualitative and semiquantitative discrimination of distinct product patterns [4]. Four such pattern types were obtained: (i) cellobiohydrolases (CBHs), (ii) non-processive endoglucanases without predominant hydrolysis products (EGs), apparent processive endoglucanases with (iii) cellotetraose as the intermediate product (pEG4), and (iv) cellobiose as the major product during substrate hydrolysis (pEG2).

Cellulosomal GH9 proteins were shown to produce all four types of cellulase product patterns and seem to be the most diverse enzyme family with regard to composition of the module architecture, product spectrum, and activity mode (Figs. 1, 2). Cbh9A, Cel9K, and Cel48S are CBHs, specifically releasing cellobiose from unmodified cellulose and cellodextrins, whereas they do not efficiently hydrolyze CMC and mixed-linkage β-glucan from barley (Figs. 1, 2). The processive action of CBHs, leading to the release of cellobiose, is favored by the 180° rotation of the glucose moieties within the cellulose chain [4]. Non-processive endo-acting β-1,4-glucanases (EGs) are characterized by their indiscriminate scission of cello-oligosaccharides and an acceptance of substrates with side chain modifications or mixed-linkage substrates. Thin-layer chromatographic product analysis revealed that this endoglucanase group generates cellodextrins with no preferential hydrolysis pattern when tested on the different types of substrates. Three GH families were found to show this type of endoglucanase activity, with the highest activities seen on CMC and barley β-glucan, namely GH5 (Cel5E and Cel5H), GH8 (Cel8A), and GH9 proteins (Cel9-44J and Cel9T). The results from TLC analysis support this finding, as long-chain products (e.g., cellopentaose or larger, DP ≥ 5) which are characteristic to non-processive endoglucanases were observed in their digestion patterns.

In contrast, processively acting endoglucanases regularly show low activity on CMC and barley β-glucan. This can be explained by steric hindrance inhibiting further substrate cleavage, or by immobilization of the enzymes as carbohydrate-binding modules inhibit dissociation from the tightly bound substrate. Interestingly, about half of the cellulosomal endoglucanases produce cellotetraose as the intermediate product (i.e., pEG4-type cellulases: Cel9F, Cel9N, Cel9P, Cel9Q, Cel9R, Cel9T, Cel9U, Cel9V, Cel9W, Lec9A, and Lec9B). With the exception of Cel9P, they all share identical module architecture with a GH9 catalytic module connected to a CBM3c. A major functional role of the CBM is to decrease the enzyme dissociation constant *k*_off_ by interaction of the polysaccharide chain with a diverse set of binding residues on the CBM surface [5]. In processive endoglucanases, the catalytic module is joined to a family 3c carbohydrate-binding module that is aligned with the active site cleft. The endoglucanase Cel9A from *T. fusca* was shown to be processive upon the presence of the CBM3c module, whereby the truncation of the binding module converted the enzyme into a non-processive endoglucanase [5, 59]. In terms of bioenergetics it seems reasonable to infer that *C. thermocellum* expresses a redundant and large set of processive endoglucanases, as cellotetraose was shown to be preferably assimilated during growth on cellulose [64].

The most interesting observation of this study was the detection of cellobiose as main product of the pEG2-type cellulases, which was found in endoglucanase GH5 sub-family 1 proteins (Cel5B, Cel5G, Cel5L, Cel5O) and one representative of GH9 (Cel9D). Cel5O is the only representative of cellobiose-producing endoglucanases of type pEG2 that comprises a CBM3b module. In this study, Cel5O shows characteristics of a processively active endoglucanase rather than the suggested cellobiohydrolase function that has been reported previously [36].

Of particular note is that a mixture of non-processive and processive-type endoglucanases within a nonavalent complex (*n* = 9 different enzymes, currently named “all EG types”), which reconstitutes the intricate cellulosome, achieved the most efficient degradation of cellulose with a recombinant enzyme complex in this study (Fig. 3a, b). A native enzyme mixture from the *cipA*-deficient *C. thermocellum* mutant SM901 [41] complexed with recombinant CipA8 reached almost the same activity as the native cellulosomal complex. These data are in accordance with previously published results, where a higher cellulolytic efficiency was observed with a more diverse complex composition [43, 63, 65, 66]. The observed diversity of the hydrolysis pattern and substrate specificity of the cellulosomal cellulases may be an adaptation of the cellulosome complex to avoid stalling (also referred as jamming) of cellulases during substrate degradation [67]. Our results therefore indicate that different endoglucanase types present in the cellulosome complex may contribute to its high efficiency in lignocellulosic biomass degradation.

Sequence and structural comparison of cellulosomal GH9 cellulases allow identifying binding residues that may interact with cello-oligosaccharide sugar moieties entering the catalytic cleft upon hydrolysis (Fig. 5; Additional file 7). The (α/α)_6_-barrel fold of *T. fusca* cellulase Cel9A, a cellobiose-producing enzyme, contains an open active site cleft and at least 9 sugar-binding subsites to bind positions + 4 to − 2 [59]. The lack of substrate-binding residues from subsites − 1 to − 4 results in weaker binding. The dissociation of the sugar chain bound to the enzyme rather than entering the empty subsites after cleavage results in decreased cellulase processivity [5, 33]. In the cellulosomal GH9 cellulases, comprising most of the pEG4 enzymes with cellotetraose as the intermediate product, conserved aromatic and electrically charged residues were identified that may correlate with the observed product formation pattern: non-processive endoglucanases and pEG4 comprise additional tryptophan or tyrosine residues that were shown to bind the − 3 and − 4 sugar moieties, and are absent in the CBHs Cel9K and Cbh9A and in the pEG2 enzyme Cel9D. These additional binding subsites may explain the production of longer oligosaccharide products during hydrolysis (such as DP 4) by binding a larger portion of the cellulose chain. In turn, the presence of more binding residues at the − 2 to + 2 subsites may result in an increased processivity via higher affinity to the sugar chain after cleavage. From molecular docking models, this stronger binding capacity causes conformational changes to the cello-oligosaccharide (see Additional file 7). Indeed, Cbh9A and Cel9D share two additional amino acid positions for a tighter binding of the + 1/+ 2 subsites, specifically F276 (binding + 1) and F279 (+ 2) in Cel9D and W473 (+ 1) and L476 (+ 2) in Cbh9A, respectively. These amino acid residues are absent in the other cellulase types (pEG4 and EG) and may trigger the release of cellobiose as the main hydrolysis product (+ positions are the subsites of an enzymatically bound sugar chain that are released as products after hydrolysis).

Strikingly, structural similarities were also found. Cbh9A and Cel9D both share an immunoglobulin-like module that was shown to stabilize the catalytic module in Cbh9A [11]. In another study, the effect of a N-terminal extremity of Cel5F from *S. degradans* was shown to protrude into the active site of the neighboring enzyme within a trimeric quaternary structure [13], thereby influencing the substrate specificity of the cellulase. Although Cbh9A exhibits a higher sequence similarity with Cel9D than to the other cellulases (29% amino acid identity) and a similar product spectrum, Cel9D lacks the characteristic loop structure from Cbh9A, which blocks the active site after the − 2 subsite [56], thus allowing the initial endo-attack of Cel9D. Cel9D comprises less binding residues than Cbh9A which leads to a lower binding affinity for the substrate as shown by molecular docking analysis (Additional file 7). This could be due to the structure of the catalytic cleft which is flatter and broader in Cel9D than for the other glycoside family GH9 proteins.

## Conclusions

From a comparative analysis of all 24 cellulosomal β-1,4-glucanases from *C*. *thermocellum*, four different product formation patterns are observed that coincide with the apparent processivity of these enzymes. The data suggest that the presence of each processivity type is necessary for peak complex activity and therefore contributes to the high efficiency of the cellulosome. Our study paves the way for the future optimization of cellulosomal complexes by supporting a deeper understanding of the synergistic action of cellulases of different processivity types. These results may help to target efficient enzyme mixtures for industrial degradation of lignocelluloses as a basis for second generation biofuels.

## Additional files

**Additional file 1.** Primer sequences used in this study.

**Additional file 2.** Schematic representation of functional modules and primary amino acid sequence of synthesized scaffolding protein A from *C. thermocellum*.

**Additional file 3.** SDS-PAGE gel documentation of all proteins used in this study after the purification process.

**Additional file 4.** Binding properties of cellulosomal cellulases on the recombinant scaffolding protein CipA8.

**Additional file 5.** Product degradation pattern of glucose tetramers with selected cellulosomal cellulases using HPAED-PAD.

**Additional file 6.** Purification and characterization of native cellulosome complex from *C. thermocellum*.

**Additional file 7.** Molecular docking of cellohexaose in the catalytic cleft of selected cellulosomal cellulases.

## Abbreviations

CBH: cellobiohydrolase
CBM: carbohydrate-binding module
CMC: carboxymethylcellulose
Coh: cohesin
DP: degree of polymerization
EG: endoglucanase (endo-mode cellulases with no specific hydrolysis pattern)
EMSA: gel mobility shift assay
GH: glycoside hydrolase
HPAEC-PAD: high-performance anion-exchange chromatography with pulsed amperometric detection
PASC: phosphoric acid swollen cellulose
pEG2: processive endoglucanases with cellobiose as the main product
pEG4: processive endoglucanases with cellotetraose as intermediate product
SDS-PAGE: sodium dodecyl sulfate–polyacrylamide gel electrophoresis
TCEP: tris (2-carboxyethyl) phosphine
TLC: thin-layer chromatography
