# Supplementary material for: Comparative characterization of all cellulosomal cellulases from Clostridium thermocellum reveals high diversity in endoglucanase product formation essential for complex activity
Source: Biotechnol Biofuels. 2017 Oct 23;10:240. doi: 10.1186/s13068-017-0928-4 (PMC5651568; doi:10.1186/s13068-017-0928-4)
Supplement: Supplementary file 1 — Additional file 1. Primer sequences used in this study. [file 13068_2017_928_MOESM1_ESM.docx]

**Additional file 1:** Primer sequences used in this study.

| Gene | Primer name | sequence (5' to 3') |
| --- | --- | --- |
| *cel9N* | Cel9N-fw | GATCGAGCTCTCTTCGCCTCGTTACGGCG |
|  | Cel9N-rev | GATCCTCGAGTATAGGTAACGAACCAATTAACT |
| *cel5-26H* | Cel5-26H-fw | GATCGGATCCTACAACAGTGGTTTAAAAATCGG |
|  | Cel5-26H-rev | GATCCTCGAGTATGGGTATTTCACTGATGGC |
| *cel9P* | Cel9P-fw | GATCGGATCCGATTATGCCACCGCATTAAAAT |
|  | Cel9P-rev | GATCCTCGAGATTTATGATGTTTCCATAGATATC |
| *cel9D* | Cel9D-fw | GATCGGATCCGAGACCAAAGTGTCAGCTGC |
|  | Cel9D-rev | GATCCTCGAGTATTGGTAATTTCTCGATTACCC |
| *cel9V* | Cel9V-fw | GATCGAGCTCGCACCACCTGCGACTTTTAC |
|  | Cel9V-rev | GATCCTCGAGAAAAGACGTTATTATGCCAAGAA |
| *cel9U* | Cel9U-fw | GATCGAGCTCGCCGAACCGGAATATAATTTTG |
|  | Cel9U-rev | GATCCTCGAGCTTTACGGGCAGTTTTTCTATT |
| *cel5L* | Cel5L-fw | GATCGGATCCGATCCGAACAATGACGACTG |
|  | Cel5L-rev | GATCCTCGAGTATTGGTATTTTAAGCACTTTCC |
| *lec9A* | Lec9A-fw | GATCGGATCCGAGCCAAAATTTAACTATGTAGA |
|  | Lec9A-rev | GATCCTCGAGCTGCTCTACGGGGAACTTAT |
| *lec9B* | Lec9B-fw | GATCGAGCTCAATGTGGAATACAACTATGCAAA |
|  | Lec9B-rev | GATCCTCGAGTTTTATCGGAATAACCTCGATTG |
| *cel5O* | Cel5O-fw | GATCGGATCCGACACTTCTGAAGAACCCGC |
|  | Cel5O-rev | GATCCTCGAGTTCATTTTGGTTTTCTTCCACC |
| *cbh9A* | Cbh9A-fw | GATCGAGCTCGAAGATAATTCTTCGACTTTGC |
|  | Cbh9A-rev | GATCCTCGAGTCGATATGGCAATTCTTCTATG |
| *cel5B* | Cel5B-fw | GATCGGATCCGAAGGGTCATATGCTGATTTG |
|  | Cel5B-rev | GATCCTCGAGTTTATACGGCAACTCACTTATG |
| *cel5E* | Cel5E-fw | GATCGGATCCTCACCGGTAAAAGGCTTTCAG |
|  | Cel5E-rev | GATCCTCGAGGCCCGGATTGTCTTCATCAG |
| *cel5G* | Cel5G-fw | GATCGGATCCAATACCGGTTCAACAGCTACG |
|  | Cel5G-rev | GATCCTCGAGGGTGGTGTGCGGCAGTTTG |
| *cel9F* | Cel9F-fw | GATCGAGCTCGATTTCAACTATGGTGAGGCA |
|  | Cel9F-rev | GATCCTCGAGCTGTTCAGCCGGGAATTTTTC |
| *cel9R* | Cel9R-fw | GATCGGATCCGACTATAACTATGGAGAAGCAC |
|  | Cel9R-rev | GATCCTCGAGTGAATTTCCGGGTATGGTTGG |
| *cel9T* | Cel9T-fw | GATCGGATCCGAATACAATTATGCAAAGGCGC |
|  | Cel9T-rev | GATCCTCGAGTATAGGGAGAGACGGTATGC |
| *cel9W* | Cel9W-fw | GATCGGATCCACTACATTCAACTACGGAGAAG |
|  | Cel9W-rev | GATCCTCGAGAGGTGCGTAAGGCAGTTTGC |
| *cel8A* | Cel8A-fw | AGCGGCAGGTGAGCTCTTTAA |
|  | Cel8A-rev | GTAGGTGGTCGACGCTCTTTAT |
| *celQ* | CelQ-fw | GATCAAGCTTTGCTGACGGCATTTATTCTTCC |
|  | CelQ-rev | GATCCTCGAGTACCGGAAATTTATCTATTATACGG |
| *bglT* | bglT-fw | GATCGAATTCATGACCGAGAACGCCGAAAA |
|  | bglT-rev | GATCAAGCTTGGTCTGGGCCCGCGCG |
