# Supplementary material for: Comparative characterization of all cellulosomal cellulases from Clostridium thermocellum reveals high diversity in endoglucanase product formation essential for complex activity
Source: Biotechnol Biofuels. 2017 Oct 23;10:240. doi: 10.1186/s13068-017-0928-4 (PMC5651568; doi:10.1186/s13068-017-0928-4)
Supplement: Supplementary file 2 — Additional file 2. Schematic representation of functional modules and primary amino acid sequence of synthesized scaffolding protein A from C. thermocellum. [file 13068_2017_928_MOESM2_ESM.docx]

**Additional file 2:** Schematic representation of functional modules and primary amino acid sequence of synthesized scaffolding protein A from *C. thermocellum*. The CipA8 enzyme sequence is identical to WP_020458017.1 lacking the cohesin module on position 6 (Coh6) and the C-terminal dockerin type-II (Doc-II).


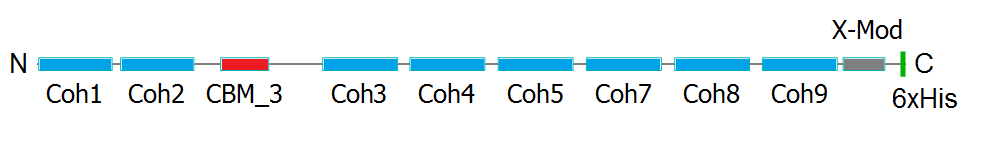


>CipA8

MATMTVEIGKVTAAVGSKVEIPITLKGVPSKGMANCDFVLGYDPNVLEVTEVKPGSIIKDPDPSKSFDSAIYPDRKMIVFLFAEDSGRGTYAITQDGVFATIVATVKSAAAAPITLLEVGAFADNDLVEISTTFVAGGVNLGSSVPTTQPNVPSDGVVVEIGKVTGSVGTTVEIPVYFRGVPSKGIANCDFVFRYDPNVLEIIGIDPGDIIVDPNPTKSFDTAIYPDRKIIVFLFAEDSGTGAYAITKDGVFAKIRATVKSSAPGYITFDEVGGFADNDLVEQKVSFIDGGVNVGNATPTKGATPTNTATPTKSATATPTRPSVPTNTPTNTPANTPVSGNLKVEFYNSNPSDTTNSINPQFKVTNTGSSAIDLSKLTLRYYYTVDGQKDQTFWCDHAAIIGSNGSYNGITSNVKGTFVKMSSSTNNADTYLEISFTGGTLEPGAHVQIQGRFAKNDWSNYTQSNDYSFKSASQFVEWDQVTAYLNGVLVWGKEPGGSVVPSTQPVTTPPATTKPPATTKPPATTIPPSDDPNAIKIKVDTVNAKPGDTVNIPVRFSGIPSKGIANCDFVYSYDPNVLEIIEIKPGELIVDPNPDKSFDTAVYPDRKIIVFLFAEDSGTGAYAITKDGVFATIVAKVKSGAPNGLSVIKFVEVGGFANNDLVEQRTQFFDGGVNVGDTTVPTTPTTPVTTPTDDSNAVRIKVDTVNAKPGDTVRIPVRFSGIPSKGIANCDFVYSYDPNVLEIIEIEPGDIIVDPNPDKSFDTAVYPDRKIIVFLFAEDSGTGAYAITKDGVFATIVAKVKSGAPNGLSVIKFVEVGGFANNDLVEQKTQFFDGGVNVGDTTEPATPTTPVTTPTTTDDLDAVRIKVDTVNAKPGDTVRIPVRFSGIPSKGIANCDFVYSYDPNVLEIIEIEPGDIIVDPNPDKSFDTAVYPDRKIIVFLFAEDSGTGAYAITKDGVFATIVAKVKSGAPNGLSVIKFVEVGGFANNDLVEQKTQFFDGGVNVGDTTEPATPTTPVTTPTTTDDLDAVRIKVDTVNAKPGDTVRIPVRFSGIPSKGIANCDFVYSYDPNVLEIIEIEPGELIVDPNPTKSFDTAVYPDRKMIVFLFAEDSGTGAYAITEDGVFATIVAKVKSGAPNGLSVIKFVEVGGFANNDLVEQKTQFFDGGVNVGDTTEPATPTTPVTTPTTTDDLDAVRIKVDTVNAKPGDTVRIPVRFSGIPSKGIANCDFVYSYDPNVLEIIEIEPGDIIVDPNPDKSFDTAVYPDRKIIVFLFAEDSGTGAYAITKDGVFATIVAKVKEGAPNGLSVIKFVEVGGFANNDLVEQKTQFFDGGVNVGDTTVPTTSPTTTPPEPTITPNKLTLKIGRAEGRPGDTVEIPVNLYGVPQKGIASGDFVVSYDPNVLEIIEIEPGELIVDPNPTKSFDTAVYPDRKMIVFLFAEDSGTGAYAITEDGVFATIVAKVKEGAPEGFSAIEISEFGAFADNDLVEVETDLINGGVLVTNKPVIEGYKVSGYILPDFSFDATVAPLVKAGFKVEIVGTELYAVTDANGYFEITGVPANASGYTLKISRATYLDRVIANVVVTGDTSVSTSQAPIMMWVGSDYDAQDPNSSSVDKLAAALEHHHHHH
