# Supplementary material for: Comparative characterization of all cellulosomal cellulases from Clostridium thermocellum reveals high diversity in endoglucanase product formation essential for complex activity
Source: Biotechnol Biofuels. 2017 Oct 23;10:240. doi: 10.1186/s13068-017-0928-4 (PMC5651568; doi:10.1186/s13068-017-0928-4)
Supplement: Supplementary file 3 — Additional file 3. SDS-PAGE gel documentation of all proteins used in this study after the purification process. [file 13068_2017_928_MOESM3_ESM.docx]

**Additional file 3:** SDS-PAGE gel documentation of all proteins used in this study after the purification process (IMAC purification and heat precipitation). The protein amount was 0.5 to 1.0 µg per lane.


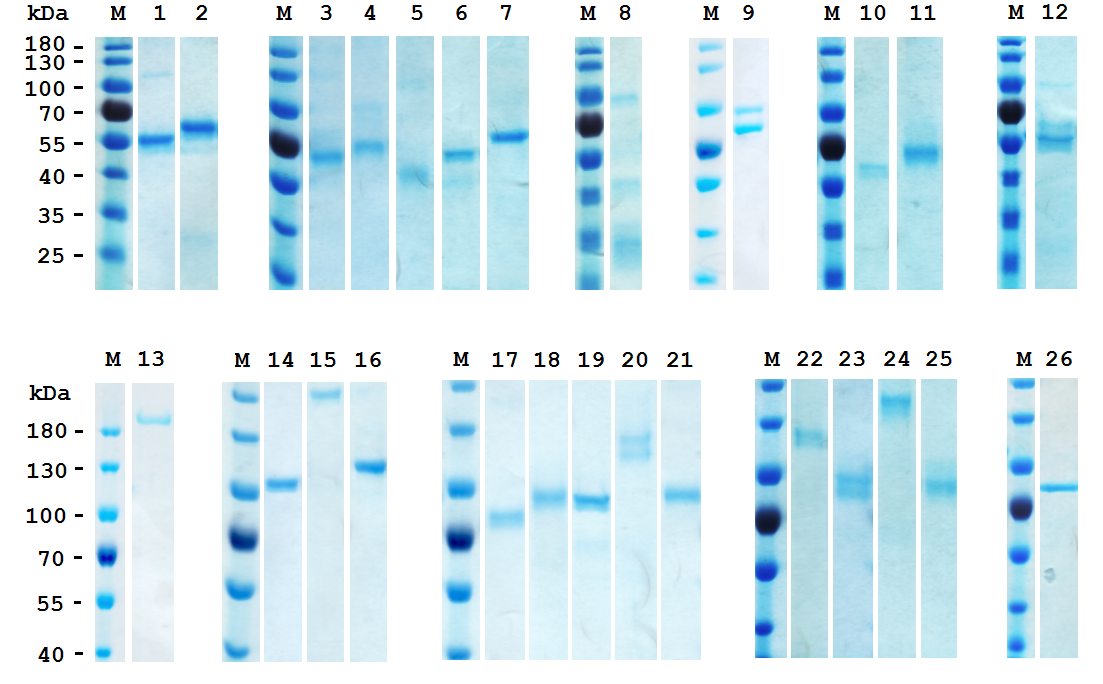


| **lane** | **description** | **expected size (kDa)** |
| --- | --- | --- |
| M | Marker (Thermo Scientific) |  |
| 1 | Cel8A | 51.8 |
| 2 | Cel5L | 59.2 |
| 3 | Cel5B | 63.5 |
| 4 | Cel9D | 71.1 |
| 5 | Cel5E* | 54.4 |
| 6 | Cel5G | 62.9 |
| 7 | Cel5O | 74.1 |
| 8 | Cel124A | 37.4 |
| 9 | Lec9A | 80.1 |
| 10 | Cel9P | 61.9 |
| 11 | Cel9T | 67.3 |
| 12 | *Thermus thermophilus* BglT | 51.8 |
| 13 | Recombinant scaffolding protein CipA8 | 172.4 |
| 14 | Cel5-26H | 102.0 |
| 15 | Cel9-44J | 176.2 |
| 16 | Cel9K | 100.4 |
| 17 | Cel9Q | 81.7 |
| 18 | Cel9R | 81.7 |
| 19 | Cel48S | 83.8 |
| 20 | Cel9U | 106.2 |
| 21 | Cel9N | 80.9 |
| 22 | Cel9V | 107.8 |
| 23 | Cel9W | 81.7 |
| 24 | Cbh9A | 137.7 |
| 25 | Lec9B | 88.5 |
| 26 | Cel9F | 82.2 |

* Cel5E was expressed without carbohydrate esterase module
