# Supplementary material for: Comparative characterization of all cellulosomal cellulases from Clostridium thermocellum reveals high diversity in endoglucanase product formation essential for complex activity
Source: Biotechnol Biofuels. 2017 Oct 23;10:240. doi: 10.1186/s13068-017-0928-4 (PMC5651568; doi:10.1186/s13068-017-0928-4)
Supplement: Supplementary file 4 — Additional file 4. Binding properties of cellulosomal cellulases on the recombinant scaffolding protein CipA8. [file 13068_2017_928_MOESM4_ESM.docx]

**Additional file 4:** Binding properties of cellulosomal cellulases on the recombinant scaffolding protein CipA8 (8 cohesin binding sites).

| **enzyme** | **Binding stoichiometry**  **(CipA8 : cellulase)** |
| --- | --- |
| Cel8A | 1:4 |
| Cel5L | 1:8 |
| Cel5B | 1:6 |
| Cel9D | 1:8 |
| Cel5E* | 1:8 |
| Cel5G | 1:8 |
| Cel5O | <1:4 |
| Cel124A | n.d. |
| Lec9A | 1:8 |
| Cel9P | ≥1:4* |
| Cel9T | 1:8 |
| Cel5-26H | 1:6 |
| Cel9-44J | 1:6 |
| Cel9K | 1:8 |
| Cel9Q | 1:6 |
| Cel9R | 1:8 |
| Cel48S | 1:6 |
| Cel9U | 1:2 |
| Cel9N | 1:8 |
| Cel9V | 1:6 |
| Cel9W | 1:8 |
| Cbh9A | 1:8 |
| Lec9B | 1:8 |
| Cel9F | <1:4 |

n.d.: binding could not be determined, due to low amount of enzyme

*: Too low amount of enzyme to test higher molar ratios
