# Supplementary material for: Comparative characterization of all cellulosomal cellulases from Clostridium thermocellum reveals high diversity in endoglucanase product formation essential for complex activity
Source: Biotechnol Biofuels. 2017 Oct 23;10:240. doi: 10.1186/s13068-017-0928-4 (PMC5651568; doi:10.1186/s13068-017-0928-4)

**Additional file 5:** Product degradation pattern of glucose tetramer type B (G4G3G4G) and type C (G4G4G3G) with selected cellulosomal cellulases using HPAED-PAD. Oligosaccharide markers: Laminarin-oligosaccharides with DP 1 - 3 (L 1-3); Cello-oligosaccharide standards with DP 1 - 6 (C1-6). The first peak represents the injection peak whereas the distinct peak at approx. 11 min is due to sucrose added as cryopreservative in the enzymatic preparations (see Materials and Methods section).
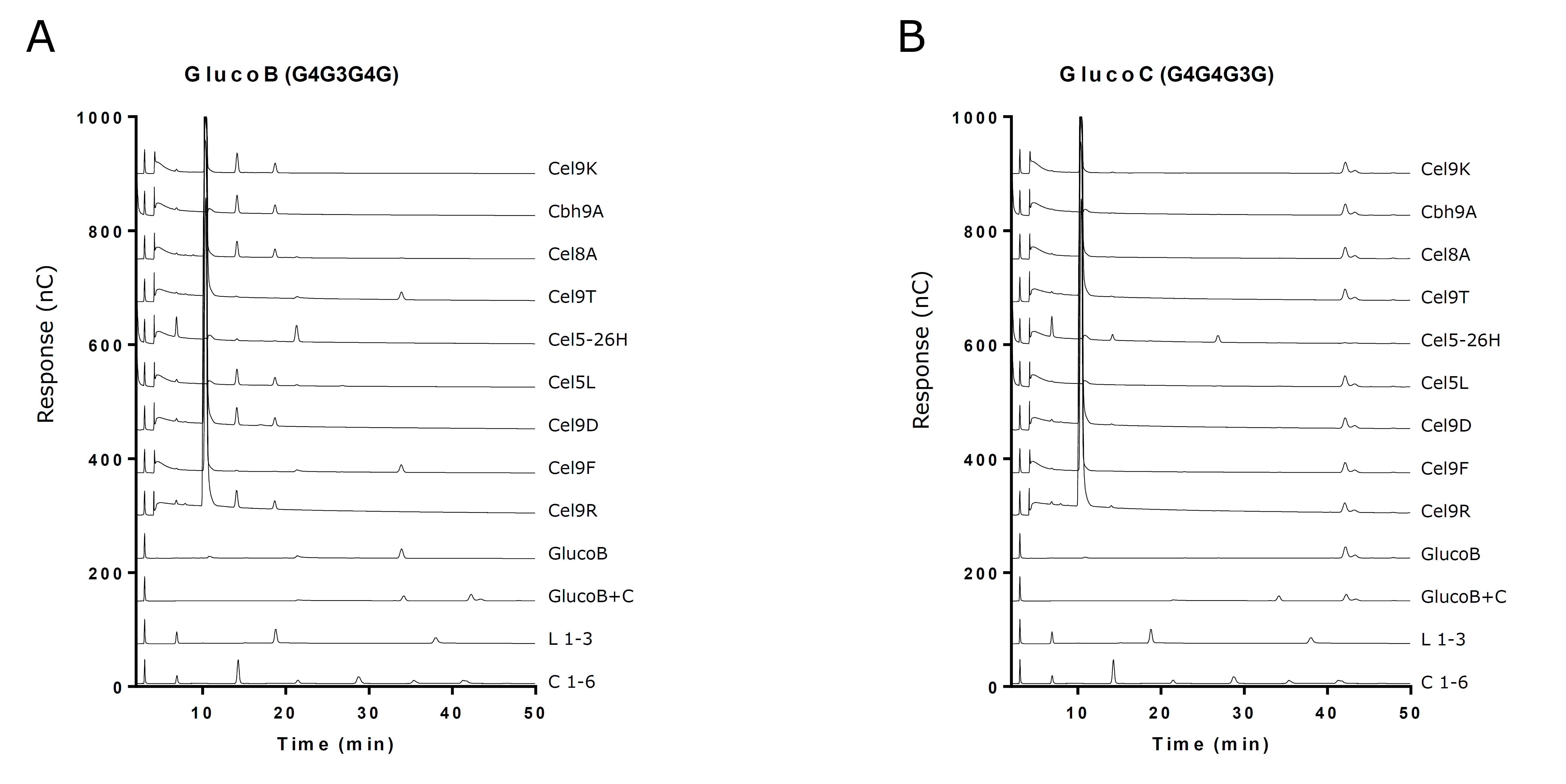

Supplement: Supplementary file 5 — Additional file 5. Product degradation pattern of glucose tetramers with selected cellulosomal cellulases using HPAED-PAD. [file 13068_2017_928_MOESM5_ESM.docx]
