# Supplementary material for: Comparative characterization of all cellulosomal cellulases from Clostridium thermocellum reveals high diversity in endoglucanase product formation essential for complex activity
Source: Biotechnol Biofuels. 2017 Oct 23;10:240. doi: 10.1186/s13068-017-0928-4 (PMC5651568; doi:10.1186/s13068-017-0928-4)
Supplement: Supplementary file 6 — Additional file 6. Purification and characterization of native cellulosome complex from C. thermocellum. [file 13068_2017_928_MOESM6_ESM.docx]

**Additional file 6:** Purification and characterization of native cellulosome complex from *C. thermocellum*. A: Anaerobic flasks with GS2 growth media containing a strip of filter paper without (-) and with (+) *C. thermocellum* inoculum after 2 days of incubation at 60 °C. The activity of the cellulosome is visible by the degradation of the filter paper (+), whereas native cellulosome is prepared after complete substrate hydrolysis from the culture supernatant. B: 8 % SDS-PAGE control of native cellulosome preparation (1: protein marker; 2: native cellulosome). C: Temperature maximum of the native cellulosome after 20 hours of incubation on 1 % Avicel. D: Determination of pH optimum after 24 hours of incubation on 1 % Avicel at 60 °C. Buffers were sodium acetate (pH 4 - 5.5), MES buffer (pH 5.5 - 6.5), MOPS buffer (pH 6.5 - 7.5) and TRIS buffer (pH 7.5 - 9.0).
