# Supplementary material for: Comparative characterization of all cellulosomal cellulases from Clostridium thermocellum reveals high diversity in endoglucanase product formation essential for complex activity
Source: Biotechnol Biofuels. 2017 Oct 23;10:240. doi: 10.1186/s13068-017-0928-4 (PMC5651568; doi:10.1186/s13068-017-0928-4)
Supplement: Supplementary file 7 — Additional file 7. Molecular docking of cellohexaose in the catalytic cleft of selected cellulosomal cellulases. [file 13068_2017_928_MOESM7_ESM.docx]

**Additional file 7:** A: Molecular docking of cellohexaose (represented as stick model) in the catalytic cleft of selected cellulosomal cellulases (grey), shown as surface model. The modelling parameters are summarized in the materials section. B: From molecular docking experiments, the best 20 pose models were selected for the calculation of the simulated binding affinity (kcal/mol) of cellohexaose with the different cellulases and the results are depicted as box-plots.
